# Human-induced temperature rise is driving Africa towards drought-prone climatic conditions

**DOI:** 10.1038/s41598-025-34010-6

**Published:** 2026-01-03

**Authors:** Basudev Swain, Marco Vountas, Rui Song, Aishwarya Singh, Vittal Hari, Md Saquib Saharwardi, Akshaya Nikumbh, Adrien Deroubaix, Pritanjali Shende, Luca Lelli, Richard Alawode, Sachin S. Gunthe

**Affiliations:** 1 Department of Physics, Atmospheric, Oceanic and Planetary Physics, University of Oxford, Oxford, UK; 2 Institute of Environmental Physics, University of Bremen, Bremen, Germany; 3 School of Earth and Atmospheric Sciences, Georgia Institute of Technology, Atlanta, GA United States; 4 Department of Environmental Science and Engineering, Indian Institute of Technology (ISM) Dhanbad, Dhanbad, India; 5 Physical Science and Engineering Division, King Abdullah University of Science and Technology, Thuwal, Kingdom of Saudi Arabia; 6 Department of Climate Studies, Indian Institute of Technology Bombay, Maharashtra, India; 7 Max-Planck-Institut für Meteorologie, Hamburg, Germany; 8 Indian Institute of Tropical Meteorology, Pune, India; 9 Remote Sensing Technology Institute, German Aerospace Centre (DLR), Wessling, Germany; 10 Leipziger Institut für Meteorologie, Leipzig University, Leipzig, Germany; 11 Centre for Atmospheric and Climate Sciences, Indian Institute of Technology Madras, Chennai, India

**Keywords:** Climate sciences, Atmospheric science, Atmospheric dynamics

## Abstract

This study focuses on the role of human activities in shaping climate forcings and their impact on surface air temperature (SAT) and drought intensification over Africa, emphasizing the human contributions to these phenomena. Through the analysis of observations, various model experiments, and Regularized Optimal Fingerprinting detection technique, our findings indicate that human-induced factors have contributed to an increase in surface air temperatures ranging from 0.8 to $1.06^{\circ }$C above pre-industrial benchmarks. Greenhouse gases (GHGs) emerge as the primary driver of this rise (0.47 to $0.92^{\circ }$C), followed by land use (LU) changes (0.47 to $0.63^{\circ }$C). In contrast, anthropogenic aerosols (Aaer) exert a cooling effect (-1.82 to $-1.36^{\circ }$C) on SAT. The analysis reveals that SAT anomalies, particularly during the industrial period, have significantly contributed to the intensification of drought-prone climatic conditions. During the pre-industrial period, the absence of anthropogenic warming kept SAT stable, resulting in mildly wet conditions (Standardized Precipitation Evapotranspiration Index (SPEI)=0.54). However, in the industrial period, the sharp rise in SAT due to GHG and LU forcings led towards significantly drought-prone climatic conditions (SPEI=-0.73), while the cooling effect of Aaer was insufficient to offset the warming trend. Estimates based on Representative Concentration Pathways (RCP) 4.5 and 8.5 suggest that the SAT over Africa could rise by around $2^{\circ }$C and $5^{\circ }$C, respectively, by the end of the century, highlighting the significant influence of human-driven factors in driving temperature rise. Strategic oversight of GHG emissions, LU changes, and aerosol concentrations in Africa offers the possibility potential to mitigate further warming and consequent drought intensification in this region.

## Introduction

The rise in surface air temperature (SAT) over Africa in recent decades has become increasingly pronounced^1,2^, leading to more frequent droughts^3,4^, which have a range of impacts including altered rainfall patterns^5^, intensified heat waves^6^, threatened agricultural productivity^7^, and amplified food and water insecurity among vulnerable populations^7^. Addressing these challenges requires coordinated efforts at local, national, and international levels, prioritizing the understanding and mitigation of both natural and anthropogenic factors contributing to rising SAT. Such efforts are essential to ensure a more sustainable and resilient future for Africa, particularly facing increasingly frequent drought conditions.

Different areas experience unique temperature changes compared to the worldwide mean^8^, and local warming is driven by various human-induced factors specific to those regions^9^. Thus, comprehending the role of human activity in driving SAT and its causal link to rising drought conditions in Africa is crucial for several reasons. Most studies on anthropogenic influences on SAT have focused on the Global North^9–17^, leaving Africa one of the most climate vulnerable regions understudied in terms of the contribution of human factors like greenhouse gas emissions, land-use changes, and aerosols to SAT increases and subsequent drought intensification. Africa’s reliance on agriculture and natural resources^18^ heightens its sensitivity to climate variability, but the lack of Africa-specific research hinders the development of targeted climate adaptation strategies. Moreover, climate drivers in Africa often differ from those in industrialized regions, making an Africa-specific focused approach is essential. Although very few studies have examined rising temperatures^5,19^ or drought conditions in Africa^20^ separately, there remains a gap in research on how increasing anthropogenic activity during the industrial era has contributed to both SAT rise and subsequent drought intensification over the continent.

This study aims to isolate human influences on SAT and assess their role in increasing drought risk, offering new insights essential for enhancing climate resilience in Africa. By examining the human influence on SAT increase and its consequential impact on drought, this study highlights Africa’s unique climate dynamics and human-climate interactions, distinct from those in more industrialized regions. This contributes to a more comprehensive understanding of rising temperature impacts across the African continent and socio-economic contexts. The investigation uses various model simulations conducted with different natural and anthropogenic activities influence across different spatial scales for the historical period of the 19th, 20th, as well as for the 21st century obtained from the Coupled Model Intercomparison Project 5 (CMIP5)^21^. The CMIP5 simulations provide the historical and future SAT changes due to the single forcings driven by Greenhouse Gas emissions (GHG), anthropogenically originated aerosols (Aaer), human-driven modification of land use (LU), and natural forcings from volcanic eruptions and solar-variability (NAT)^21^.

While the CMIP6 simulations^22^ provides data on GHG, aerosol (Aaer), and natural (NAT) forcings, CMIP5 is more suitable for studying human influence on temperature due to its inclusion of explicit LU forcing simulations. This is crucial for analyzing human-land-atmosphere interactions and their impact on temperature changes. LU data enables the quantification of how vegetation, urbanization, and agricultural practices influence surface air temperature, particularly in regions where land use changes significantly affect climate^17^. Although CMIP6 offers advancements in several areas, it does not treat LU as a separate factor^22^, limiting its ability to isolate land cover changes and their independent impact on temperature. In contrast, CMIP5 allows for the separation of land use effects from other forcings, such as greenhouse gases and aerosols, providing a clearer understanding of anthropogenic contributions to observed temperature changes. This makes CMIP5 especially valuable for our study, which requires a detailed breakdown of human and natural influences on SAT over Africa. By comparing and estimating the combined effects of these forcings on the Standardized Precipitation Evapotranspiration Index (SPEI)^23^, an indicator of drought conditions^24^, we can assess the role of anthropogenic factors in driving temperature rise and subsequent droughts in Africa.

To determine the extent of human-induced effects on rising SAT, this study employs the Regularized Optimal Fingerprinting (ROF) detection and attribution method^25^. This approach, widely utilized in detection and attribution research, has been applied across multiple regions in numerous studies conducted over different regions of Global North^9,10,17,25–27^. The ROF method, offers a robust and statistically rigorous approach for accessing the influences of individual climate forcings on SAT rise. Unlike direct differences between “All” (ANT+NAT) and individual forcing experiments, ROF accounts for overlaps, covariances, and interactions among forcings while effectively distinguishing signal from noise by comparing observed and modeled spatial-temporal patterns^25^. This method enhances sensitivity and precision, especially in regions with data sparsity, like Africa, where direct difference approaches may not be effective. By providing a nuanced, quantitative assessment of individual forcings, ROF significantly improves the depth and novelty of the analysis, ensuring reliable and insightful findings that align with the study’s objectives. By isolating the distinct influence of natural as well as anthropogenic forcings, ROF enables a deeper understanding of the drivers behind SAT changes, offering valuable insights for attribution studies.

To evaluate human-induced warming, we compare SAT changes against pre-industrial SAT baselines. Although different Holocene periods could serve as references^28^, this study adopts the 1850-1900 timeframe, aligning with the IPCC Special Report on Global Warming of $1.5^{\circ }$C^29^. This selection establishes a relevant foundation for assessing anthropogenic influences on SAT across Africa, both during the industrial era (1955-2018)^29^ and in future projections (2018-2100).

The novelty and originality of this study lie in its focus on the African continent, where comprehensive assessments of human contributions to SAT increase and their causal impact on rising drought conditions are limited. While numerous studies in the Northern Hemisphere have explored the influence of individual natural and anthropogenic forcings on SAT using CMIP5 model simulations^9,10,17,25–27^, a rigorous investigation of these drivers over Africa remains largely unexplored.

The key contributions of our research are as follows: (i) Comprehensive Analysis of Forcings, we have conducted an in-depth evaluation of various forcings–natural, anthropogenic, aerosol, land use, and greenhouse gases–that contribute to the SAT increase across Africa. This analysis utilizes the HadCRUT5 observational dataset, a range of CMIP5 climate models, and the ROF method, which has not been applied to African climate studies. This makes our work novel in a region underrepresented in climate literature. (ii) Exploration of SAT Causal Impacts on Drought, we have examined the causal influence of rising SAT and individual forcings on the SPEI and extend our future projections under RCP scenarios. This provides a multidimensional view of climate change impacts, particularly on drought, in Africa.

This research fills a critical gap by analyzing the unique climate dynamics and anthropogenic influences on SAT and drought intensification in Africa, a region with distinct climate sensitivities compared to the industrialized Northern Hemisphere. The insights gained are vital for developing tailored adaptation strategies in the African context.

## Results

### Temporal evolution of human contribution to the rising SAT

**Fig. 1:**
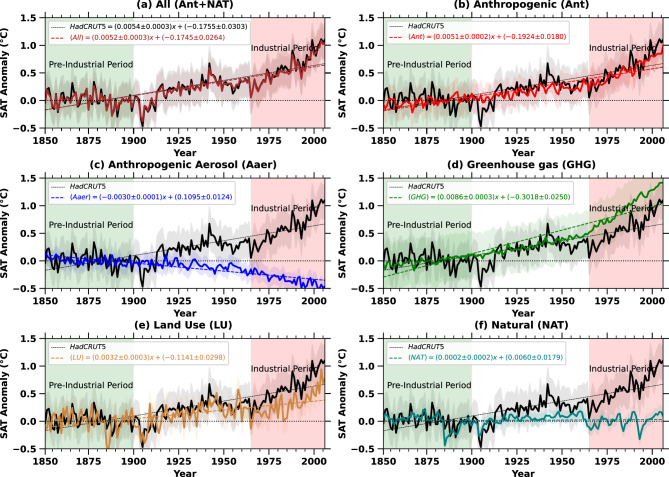
The graph presents the changing patterns of annual surface air temperature anomalies across Africa from 1860 to 2005. It includes a comprehensive category, “All,” which accounts for the combined effects of different forcings. Additionally, individual contributions from specific forcings. These forcings are Aaer, GHG, LU, natural influences (including solar incoming radiation fluctuations and volcanic activities), anthropogenic influence (Ant), defined as difference between all forcings and natural influences (Ant = All - NAT), are separately illustrated. A 95% confidence interval is considered for this analysis, with the resulting trend lines incorporated into each panel. Observational data from HadCRUT5 (represented by black lines with corresponding equations) are included in all subplots to compare modeled SAT anomalies with HadCRUT5 recorded total SAT temperature variations. Shaded regions indicate the one-standard-deviation range.

This study examines how different forcing factors have influenced SAT variations in the region from 1850 to 2005. The analysis utilizes chosen climate models alongside observational records from HadCRUT5 to assess their respective contributions over time (Fig. 1a–f). We analyzed the ensemble mean of different historical forcing factors (Ant, GHG, Aaer, LU, and NAT) for the 19th and 20th centuries, as summarized in Table S1 and illustrated in Fig. 1a–f. The temperature variations were calculated by subtracting the pre-industrial (1850-1900) annual mean SAT from the yearly averages over the study region between January 1850 and December 2005. Each panel displays shaded areas representing one standard deviation of the ensemble, along with linear regression trends. Fig. 1a highlights a consistent SAT increase from 1925 onward, marking a transition from below-zero (negative) to above-zero (positive) anomalies. This anomaly change corresponds closely with the model simulations and HadCRUT5 datasets (Fig. 1a).

A positive SAT anomaly from 1925 onwards, which indicates that the SAT is higher than a pre-industrial baseline average, is a key metric and serves as an important indicator^10^ of warming over time (Fig. 1a,b,d,e). With the exception of Aaer and NAT (Fig. 1c, f), all other forcing factors (Ant, GHG, and LU) exhibit a significant rise in SAT, reflected as a positive anomaly, beginning around 1925 (Fig. 1b, d, e). GHG forcing leads to even higher forcing than the total SAT of HadCRUT5 observations (Fig. 1d). This indicates that the combined impact of greenhouse gases (GHG) and land use (LU) alone contributes significantly more to the overall rise in surface air temperature (SAT) across Africa during the industrial era (Fig. 1d, e). At the onset of industrialization, the warming linked to GHG and LU began to escalate sharply from 1965 (Fig. 1d, e), an anomaly that aligns with both observed data and historical model projections (Fig. 1a). The influence of LU and GHG is also reflected in the anthropogenic SAT anomaly starting after 1965, in the industrial period (Fig. 1b). According to Fig. 1b, CMIP5 historical simulations show a minimal increase in anthropogenic warming before 1925, suggesting that human-induced impacts on SAT were negligible during the early part of the 20th century, with the majority of warming due to human activities occurring after 1960. Significant warming has been driven by changes in land use and GHG emissions (Fig. 1b, d, e). Additionally, the effect of anthropogenic aerosols (Aaer) is noticeable, with a clear decline in SAT beginning in the 1960s. The years 1925 and 1965 were identified through an objective analysis of SAT anomaly (Fig. 1a–f), marking significant positive shifts. These years align with key changes in climate forcings, such as 1925 corresponds to early industrialization^10,29^ and increased anthropogenic influences, while 1965 reflects post-war industrialization and a rise in greenhouse gas emissions^10,29^. These points are reflecting shifts in natural and anthropogenic climate drivers.

**Fig. 2:**
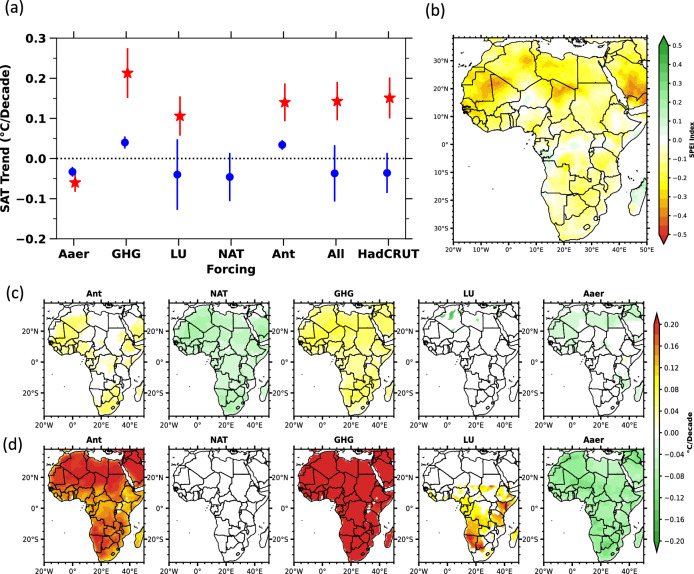
Pre-industrial and Industrial spatial SAT trends from various natural and anthropogenic forcings together with the spatial distribution of SPEI drought index. a) The SAT trends average and standard deviation of different forcing factors (such as Ant, NAT, GHG, LU, Aaer) over Africa for the pre-industrial period (in blue) and industrial period (in red), b) The spatial map of the SPEI drought index averaged over 1960 to 2018. c, d) The SAT map from different anthropogenic and natural influences are shown in top-row showing pre-industrial and in bottom-row shows the idustrial timespan respectively. The trend which is not significant are removed from the map. These maps have been generated by using Matplotlib visualization with Python version 3.10.14 (https://matplotlib.org/).

During the industrial period, the cooling effect of anthropogenic aerosols helped offset the warming caused by greenhouse gases (GHG) and land use changes (Fig. 1c). In terms of temporal trends, natural forcings had a relatively smaller impact on surface air temperature (SAT) compared to GHG and LU changes (Fig. 1f). Overall, the results suggest that aerosols played a very crucial role in reducing warming, while GHGs and land use alterations contributed substantially to the observed temperature rise over Africa (Fig. 1d, e).

In addition to examining the temporal effects of human influence, we analyzed the SAT of different forcings spatially over Africa (Fig. 2a-d). This analysis involved breaking down forcings to their individual influences, comparing average trends of SAT during the pre-industrial and industrial periods (Fig. 2a), and examining the regional distribution of both temperature and drought-like climatic conditions. For drought conditions, we used the Standardized Precipitation Evapotranspiration Index (SPEI) to assess the intensity and frequency of conditions of drought across Africa (Fig. 2b)^24^.

Observational data from HadCRUT5 and CMIP5 model ensembles indicate that during the industrial period, regions in both northern and southern Africa faced rapid rise in SAT triggered by land use ($0.13^{\circ }$C/decade) and GHG ($0.26^{\circ }$C/decade) forcings (Fig. 2d), in contrast to the time before industrialization (Fig. 2c). These SAT trends align closely with HadCRUT5, and the model simulations (Fig. S1). Across Africa, surface air temperature trends during the industrial era indicate warming driven by all forcing factors, with variations spanning from 0.03 to $0.14^{\circ }$C/decade for anthropogenic (Ant), 0.04 to $0.21^{\circ }$C/decade for GHG, and 0.04 to $0.13^{\circ }$C/decade for LU forcings. Meanwhile, natural forces (NAT) had a minimal effect (-0.04 to $0^{\circ }$C/decade), and aerosols (Aaer) induced a slight cooling (-0.03 to $-0.06^{\circ }$C/decade) (Fig. 2a). These results underscore the predominant influence of anthropogenic factors, especially GHG and LU changes, in driving SAT increases, while aerosols had a cooling influence. The impact of NAT forcing was negligible (Fig. 2a). The warming observed in the study region during the industrial era is therefore largely attributable to GHG and LU influences (Fig. 2d), consistent with observational data (Fig. S1). Additionally, the spatial distribution of the SPEI index (Fig. 2b) shows that both northern and southern Africa have experienced increasing dryness, which aligns closely with the spatial patterns of SAT increase driven by GHG emissions and land use changes (Fig. 2b, c).

### Regularized optimal fingerprinting (ROF) method: to estimate human contribution to rising SAT

The ROF method^25^ is a powerful statistical technique widely used to detect and attribute human influences on climate variables over various regions, such as SAT^9,10,17,25–27^. This method works by identifying the distinct factors of SAT rise, that are associated with specific individual forcing factors, such as greenhouse gases, aerosols, land use, or natural variability. By using the ROF method, various research has analyzed the magnitude of anthropogenic factors have influenced the observed changes in SAT over various regions of the Global North^9,10,17,25–27^. The strength of the ROF method lies in its ability to distinguish between human and natural influences, providing a robust framework for quantifying the human impact on warming trends^25^. This capability is especially valuable as it allows us to assess and compare the contributions of different forcings across various temporal scales, thus offering a nuanced understanding of the drivers behind SAT increase.

Using a combination of data from 22 different model estimations (listed in Table S1) and the HadCRUT5 dataset, along with Regularized Optimal Fingerprinting (ROF) attribution method^25^, this study evaluated the influence of anthropogenic and natural activities on the SAT rise in Africa (Table 1). The assessment shows that anthropogenic influences are responsible for an increase in SAT ranging from $0.80^{\circ }$C to $1.06^{\circ }$C (within the 5th to 95th percentile range) during the industrial period when compared to the pre-industrial era. Among the different forcings, anthropogenic aerosols (Aaer) led to a cooling of $-1.82^{\circ }$C to $-1.36^{\circ }$C, while greenhouse gases (GHG) caused a warming of $0.47^{\circ }$C to $0.92^{\circ }$C, and land use changes (LU) contributed a warming effect of $0.47^{\circ }$C to $0.63^{\circ }$C. Of these, greenhouse gases had the most significant impact on the overall rise in SAT, followed by land use changes, while natural forcings (NAT) had a minimal effect, ranging from $-0.03^{\circ }$C to $0.06^{\circ }$C. The main driver behind the rise in surface air temperatures across the study area during the industrial period is human-induced forcing, primarily due to greenhouse gas emissions, with land use changes playing the second most role.

Recently, the robustness of the optical fingerprinting method has been the subject of scientific debate. Several studies have raised concerns regarding its sensitivity to data selection, medological assumptions, and statistical interpretation^30^,[?]. Conversely, other studies^10,25^ have defended its validity when applied with appropriate constraints and rigorous calibration. This ongoing discussion underscores the importance of transparent methodology and careful evaluation when applying the optical fingerprinting approach in climate analyses.

**Table 1:** **Using ROF advanced detection methods, the impact of specific drivers on temperature shifts is calculated. The table outlines model-based assessments of temperature differences caused by various factors, expressed in Celsius, for the industrial age relative to the time before industrialization.** The $\beta$, denoting the vector of estimated coefficients, along with the 5–95% confidence ranges (shown in brackets) representing the estimated warming attributable to each factor. From the primary multimodel analysis (represented in the first row) and from HadCRUT5 data (in the second row). These estimates encompass an analysis equivalent to that of the Natural Forcings (NAT), Anthropogenic forcing (Ant), Anthropogenic Aerosols (Aaer), Greenhouse Gases forcings (GHG), and Land Use forcings (LU).

	NAT	Ant	Aaer	GHG	LU
All (Ant+NAT)	0.015 (-0.03 to 0.06)	0.93 (0.80 to 1.06)	-1.59 (-1.82 to -1.36)	0.70 (0.47 to 0.92)	0.55 (0.47 to 0.63)
HadCRUT5	0.015 (-0.04 to 0.07)	0.93 (0.80 to 1.06)	-1.62 (-1.85 to -1.40)	0.71 (0.62 to 0.80)	0.54 (0.45 to 0.63)

### Temporal causal impact of Human-induced SAT rise on drought conditions

**Fig. 3:**
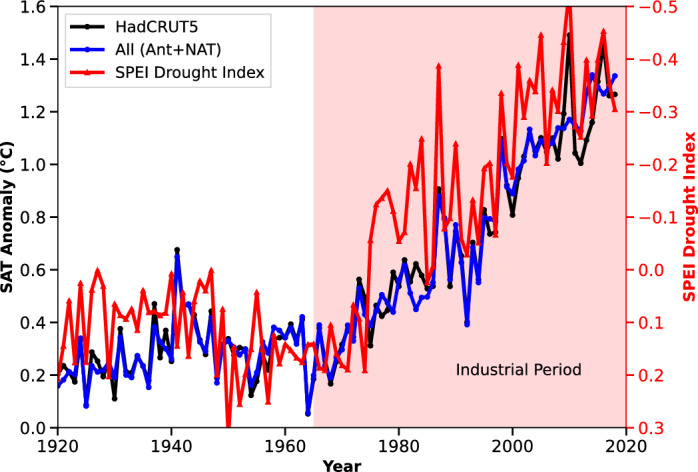
Recent industrial period surface air temperature anomaly mentioned in Fig. 1 and associated SPEI drought index from 1920 to 2018. The surface air temperature anomaly is from HadCRUT5 and multi-model average (All=Ant+NAT). As the historical model simulations from models are available up to 2005, we have considered RCP45 for the 2006 to 2018 period.

As the anthropogenic impact started from 1920 onwards (Fig. 1b), and to assess the impact of such anthropogenic contribution to drought over this study region, we used the Standardized Precipitation Evapotranspiration Index (SPEI) as an indicator^24^. Fig. 3 illustrates the temporal evolution of SAT anomalies from the observation and multi-model mean and SPEI index values over an industrial period, highlighting a significant upward trend in both parameters. The SAT anomaly graph shows a clear and persistent increase, particularly pronounced in the industrial period. This increase in SAT anomaly (1.4 $^{\circ }$C, Fig. 3) is indicative of warming over Africa, primarily driven by anthropogenic factors such as increased GHG emissions from industrial activities and fossil fuel combustion, and LU changes due to the alterations resulting from deforestation and farming activities (Fig. 1a–f). The consistent rise in SAT anomalies suggests a systematic shift in the African climate system leading to the rise in SAT, which is coinciding with the dry conditions (SPEI value of -0.5 in Fig. 3, Table 2). The SPEI index value less than zero represents dry conditions and greater than zero represents wet conditions^24^ (Table 2).

**Fig. 4:**
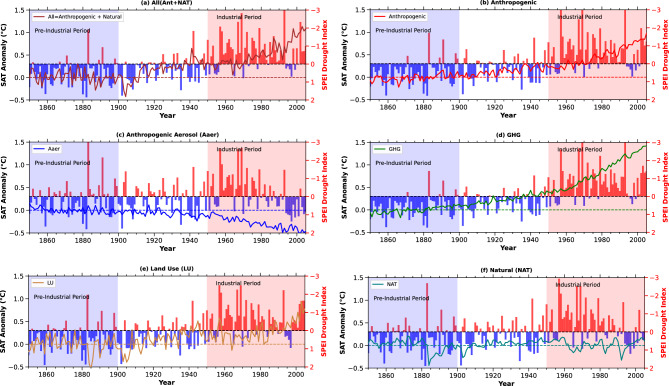
Impact of surface air temperature from specific drivers on the historical SPEI drought index. The graph shows the time-based influence of temperature changes caused by individual drivers on the SPEI drought index between 1860 and 2005. The ’All’ category combines all drivers, reflecting their combined effects. Separate drivers, including Aaer, GHG, LU, NAT (such as solar incoming radiation and volcanic events), and human-induced factors (Ant) (calculated as All minus NAT), are each represented distinctly.

**Fig. 5:**
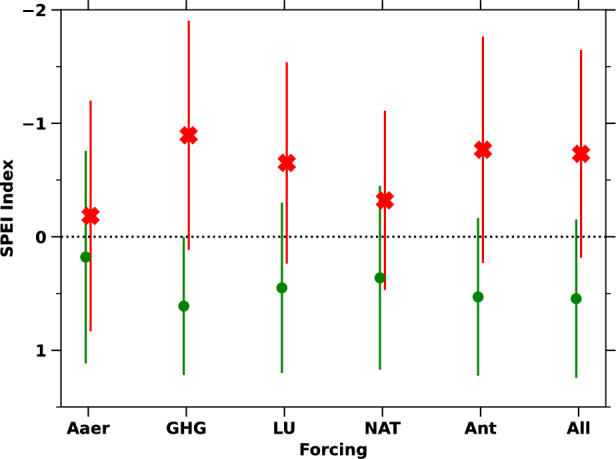
Pre-industrial (green) and Industrial (red) period causal effect of individual SAT forcings on SPEI drought index. Pre-industrial and Industrial average and one standard deviation of SPEI drought index due to various forcings forcing factors (such as Ant, NAT, GHG, LU, Aaer) over Africa for the pre-industrial period (in green) and industrial period (in red).

Simultaneously, the SPEI drought index (Table 2), which measures the severity and frequency of drought conditions^24^, exhibits a parallel upward trend (Fig. 3). This temporal alignment suggests a linkage between rising temperatures and worsening drought conditions. This is attributed to the higher SAT accelerates dry conditions^24^. These changes exacerbate drought severity and frequency^24^, particularly in vulnerable regions like Africa. The concurrent increase in temperature anomalies and drought index values underscores the impact of SAT on hydrological extremes. This is emphasizing the urgent need for a comprehensive assessment of the contribution of the total rise in SAT as well as the rise in SAT due to the individual forcings on the SPEI drought index. This will be helpful for mitigation of anthropogenic activities, and formulating strategies to address the interconnected challenges of anthropogenic warming over Africa and associated drought conditions. Thus in this study, we have assessed the causal impact of SAT due to various individual forcings on rising drought conditions over Africa over historical time period and is presented in Fig. 4a–f.

The Fig. 4(a-f) presents the temporal evolution of the annual Standardized Precipitation Evapotranspiration Index over Africa caused by SAT anomalies attributable to total and All (Ant+NAT) influence, as well as individual forcings–anthropogenic (Ant), GHG, LU, Aaer, and NAT (as per^23^, see Method section). This analysis delineates the causal effects of SAT anomalies on SPEI, highlighting their role in rising drought conditions during the industrial period compared to the pre-industrial. During the pre-industrial period, total SAT anomalies (All) were relatively stable (Fig. 4a), primarily driven by natural forcings such as volcanic aerosols and solar variability (Fig. 4a–f). This stability resulted in minor fluctuations in SPEI (Fig. 4a). The absence of higher anthropogenic warming meant that evapotranspiration rates remained close to natural levels^31^, minimizing the impact on drought conditions. SPEI values during this time serve as a critical baseline for assessing the subsequent effects of industrial-era anthropogenic forcings on Africa’s hydroclimate.

Whereas, during the industrial period, SAT anomalies increased sharply due to anthropogenic forcings (Fig. 4s–b), significantly exacerbating drought conditions across Africa (Fig. 4a–b). These rising anomalies led to a marked decline in the Standardized Precipitation Evapotranspiration Index (SPEI), shifting from an average value of 0.54 (mildly wet) in the pre-industrial period to -0.73 (mildly dry) during the industrial period (Fig. 5). The rapid rise in anthropogenic forcings transitioned Africa’s climate from near-normal conditions (SPEI = 0.52) to moderately dry conditions (SPEI = -0.76) (Figs. 4b, 5). Greenhouse gas (GHG) emissions were the primary driver of this drying trend, followed by land use (LU) changes (Figs. 4c–d, 5). These forcings rising warming, accelerating evapotranspiration, depleting soil moisture, and reducing water availability. LU changes further exacerbated drought by intensifying local warming and disrupting hydrological processes. The conversion of forests to agricultural land reduced evapotranspiration and altered precipitation patterns, compounding the drought-inducing effects of rising SAT. The combined effects of GHG and LU changes created significant regional variations in drought conditions across the continent.

In contrast, aerosol (Aaer) forcings exerted a cooling effect during the mid-20th century, partially counteracting the warming-driven declines in SPEI. Aaer helped maintain near-normal conditions in Africa throughout both the pre-industrial and industrial periods, with average SPEI values ranging from 0.17 to -0.18, respectively (Figs. 4c, 5). However, this mitigation effect was regionally variable and insufficient to offset the overall increase in drought conditions across the continent. Additionally, natural (NAT) forcings generally kept Africa’s climate in normal conditions over the historical periods (Fig. 5). This analysis of the relationship between SAT rise and drought conditions, both past and present, provides valuable insights for developing policies aimed at ensuring sustainable climate conditions for Africa’s future.

## Future projections

**Fig. 6:**
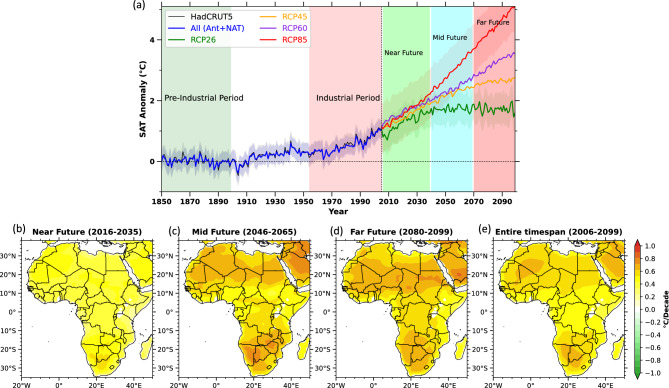
The graph illustrates historical, current, and predicted future changes in surface air temperature across Africa, both over time and space. **a** It displays the yearly average SAT deviation with respect to derived from multiple model estimations, spanning 1850-2100, compared to the baseline of 1850 to the year 1900. Future estimations, based on different Representative Concentration Pathways (RCPs), cover the time from 2006 to the year 2099. The colored shades shows one standard deviation of mean of all different CMIP5 model estimations. The HadCRUT5 (1850–2005) SAT is shown in black. The historical segment (1850–2005) is included in (a) as it is vital for contextualizing future predictions. **b**, **c**, **d**, **e** Maps show the geographical distribution of temperature trends across Africa during the 21st century under the RCP8.5 scenario. These trends are analyzed for specific intervals: the near future (2006–2035), mid-future (2046–2065), far future (2080–2099), and the entire projection period (2006–2099). These maps have been generated by using Matplotlib visualization with Python version 3.10.14 (https://matplotlib.org/).

Rising surface air temperatures across the study area, driven by combined forcings, align well with the HadCRUT5 SAT trends during the entire time span (Fig. 6a–e). The consistency between HadCRUT5 data and multi-model averages strengthens our ability to predict temperature increases in the region throughout the 21st century. Analyzing future scenarios is essential for reducing the likelihood of droughts in the area. Reducing human-driven activities presents a viable strategy to curb further temperature rises and their impact on drought conditions in Africa.

This study includes a range of future emission scenarios, from the lowest (RCP2.6) to the highest (RCP8.5) Representative Concentration Pathways. By 2100, the RCP2.6 scenario predicts a temperature increase of around $2^{\circ }$C over Africa, while RCP8.5 projects a rise of roughly $5^{\circ }$C. The overall temperature estimations under RCP8.5 show a steady upward trend, and the RCP2.6 results in an increase until mid-century, then it shows a modest decrease (Fig. 6a). Given that the RCP8.5 scenario projects the most pronounced increase in SAT across Africa, Figure 6b–e presents the spatial distribution of SAT trends over the continent during the 21st century under this high-emission pathway. The analysis encompasses four distinct time slices: the near-term (2006–2035), mid-century (2046–2065), late-century (2080–2099), and the entire projection period (2006–2099). Overall, the results indicate a marked intensification of warming over Africa as the century progresses, with substantially greater temperature increases projected for the mid- and late-century compared to the near-term. The far-future projections suggest the highest warming of about $1.2^{\circ }$C/Decade across most of the regions of Africa, except for parts of central Africa (Fig. 4c,d). The stronger warming of about 1.2 $^{\circ }$C per decade projected across most of Africa is primarily linked to soil moisture–temperature feedbacks, where reduced evapotranspiration enhances surface heating over arid and semi-arid regions. In contrast, the comparatively weaker warming in central Africa is associated with dense vegetation, high humidity, and persistent convection, which promote evaporative cooling and moderate temperature rise^32,33^. This comparative analysis across scenarios supports policymakers in prioritizing adaptation and mitigation strategies based on varying climate futures. By highlighting Africa’s unique climate sensitivities and linking SAT changes to broader climate impacts, the figure makes a crucial contribution to understanding and addressing the continent’s climate challenges.

## Discussions

The ROF approach provides valuable insights into the factors driving the rise in surface air temperature (SAT) across Africa and their effects on temperature changes. This understanding is vital for assessing the role of human activities, such as industrialization, greenhouse gas (GHG) emissions, and shifts in land use and land cover, in driving SAT increases and the resulting drought conditions. Addressing these factors is key to mitigating further impacts. While future climate shifts are expected to be largely influenced by GHG emissions, the role of LU forcing is often underestimated. Analysis conducted in this study shows a greater contributions from GHG and LU forcings, with temperature increases ranging from 0.47 to $0.92^{\circ }$C and 0.47 to $0.63^{\circ }$C, respectively, across Africa. In contrast, anthropogenic aerosols (Aaer) have a cooling effect (-1.82 to $-1.36^{\circ }$C), while natural (NAT) forcings show negligible influence.

This study also examines the causal relationship between SAT anomalies and drought conditions, comparing pre-industrial and industrial eras. During the pre-industrial period, SAT anomalies remained stable, primarily driven by natural factors, resulting in minimal SPEI variations and an average of 0.54 (indicating mildly wet conditions). In contrast, the industrial period saw a sharp rise in SAT anomalies due to human-driven factors, particularly GHG emissions, reducing SPEI to -0.73 (mildly dry) (Fig. 5). These shifts reflect a trend toward drier conditions. Land use changes exacerbated these effects by altering hydrological cycles, while aerosols provided some cooling, maintaining near-normal SPEI values of -0.18 on average. NAT forcings had little to no impact on climatic conditions.

Our findings highlight the significant role of human-induced changes, including GHG emissions, land use shifts, and aerosols, in intensifying drought conditions across Africa during the industrial era. Analyzing future scenarios is critical for mitigating droughts. Reducing human activities can help limit SAT increases and their effects on drought severity. We assessed both low (RCP2.6) and high (RCP8.5) emission scenarios, projecting temperature anomalies of roughly $2^{\circ }$C and $5^{\circ }$C by 2100, respectively. Under RCP8.5, SAT continues to climb, while RCP2.6 shows a slight decline after 2050, aligning with radiative forcing trends (Fig. 6a). Fig. 6b–e depicts spatial SAT trends under RCP8.5 for different timeframes: near future (2006–2035), mid-future (2046–2065), far future (2080–2099), and the entire projection period (2006–2099). These projections indicate significant warming, with the far future experiencing a rise of about $1.2^{\circ }$C per decade across most of Africa, except central regions (Fig. 6c,d). While similar projections exist for other regions, Africa’s SAT trends and climate impacts have received less attention. Thus, Fig. 6a–e provides detailed, Africa-specific projections under four RCP scenarios, offering high spatio-temporal resolution and historical context. This comparative analysis supports policymakers in crafting targeted adaptation strategies, emphasizing Africa’s unique climate vulnerabilities and the urgency of action.

These findings stress the need to address various human-driven factors, including GHG emissions, land use changes, and aerosols, to mitigate future SAT increases and their impact on drought conditions, fragile ecosystems, and food security in Africa. The dominance of anthropogenic forcings during the industrial era highlights the necessity for tailored reduction of anthropogenic activities over Africa.

**Table 2:** SPEI categories and corresponding climate conditions^24^.

SPEI	Climate Condition
SPEI $\ge$ 2.0	Extremely wet
1.5 $\le$ SPEI < 2.0	Severely wet
1.0 $\le$ SPEI < 1.5	Moderately wet
0.5 < SPEI < 1.0	Mildly wet
-0.5 $\le$ SPEI $\le$ 0.5	Normal
-1.0 < SPEI < -0.5	Mildly dry
-1.5 < SPEI $\le$ -1.0	Moderately dry
-2.0 < SPEI $\le$ -1.5	Severely dry
SPEI $\le$ -2.0	Extremely dry

## Data and methodology

### SAT from CMIP5 models and HadCRUT5 observation

This study assessed 22 climate models and 158 corresponding simulations spanning the 19th, 20th, and 21st centuries, as detailed in Table S1. It is important to highlight that not all simulations incorporate every type of climate forcing examined. The selected models represent various emission pathways, including RCP4.5, and account for different forcing factors: 17 simulations include NAT forcing, which is driven by changes in solar fluctuations and volcanic influences, 18 account for greenhouse gas (GHG) fluctuations, 10 simulate anthropogenic aerosol (Aaer) effects, and 5 incorporate land-use (LU) changes. These simulations, which contribute to the IPCC Fifth Assessment Report^34^, are designed to replicate climate variations over the past two centuries by integrating multiple climate drivers. Monthly mean SAT were retrieved from 22 CMIP5 model estimations for historical timespan and the RCP4.5 scenario. For the post-2005 time, the RCP4.5 pathway was used to extend the analysis through 2018, offering a realistic projection of contemporary climate conditions.

To evaluate model performance, Taylor diagrams were utilized to compare CMIP5 model-derived mean SATs with HadCRUT5 observational data^35^ across Africa from 1955 to 2005. The assessment between the HadCRUT5 observational dataset and the individual model simulations was calculated as a temporal (annual) correlation of the mean absolute surface air temperature values averaged spatially over the entire African continent for the period 1955–2005. This time frame was selected because it represents an era during which both satellite and ground-based observations were increasingly integrated into global datasets, thereby improving the reliability and consistency of temperature records. Consequently, the correlation analysis presented here captures temporal variations rather than spatial differences across the region.

All these 22 models included in this study, such as, Bcc-csm1-1, BNU-ESM, CanESM2, CCSM4, CESM1-CAM5, CNRM-CM5, CSIRO-Mk3-6-0, FGOALS-g2, GFDL-CM3, GFDL-ESM2M, GISS-E2-H, GISS-E2-R, HadGEM2-CC, HadGEM2-ES, Inmcm4, IPSL-CM5A-LR, IPSL-CM5A-MR, MIROC-ESM, MIROC5, MPI-ESM-LR, MRI-CGCM3, NorESM1-M. The Taylor diagram in Fig. S2 visualizes how well CMIP5 simulations temporally align with observational datasets. While this study utilizes only five CMIP5 models that provide simulations including land-use forcing (Table S1), this limited number reflects the restricted availability of such experiments within the CMIP5 archive rather than a methodological choice. Although a smaller ensemble may reduce the ability to fully capture the spread of model responses, the selected models still represent a diverse range of physical parameterizations and land–atmosphere interactions, offering meaningful insights into the influence of land-use changes on surface air temperature. Nevertheless, the results should be interpreted with an understanding of this constraint, and future studies would benefit from incorporating a larger ensemble of models as more land-use forcing simulations become available to further strengthen attribution assessments.

The SAT anomalies were calculated annually using area-weighted spatial averages from HadCRUT5, with anomalies referenced to the pre-industrial baseline (1850–1900). The median dataset was used to reduce uncertainties and enhance result reliability. The choice between the mean and median significantly affects the representation of ensemble-based climate anomalies. While the mean is sensitive to outliers and can exaggerate interannual variability when some models deviate strongly, the median offers a more robust central estimate by reducing the influence of extreme values. This approach minimizes model bias and provides a smoother, more representative depiction of the ensemble climate signal^36^. To ensure consistency across different datasets, SAT outputs from multiple models and simulations were re-gridded (bilinear interpolation) to a $1^{\circ }$ $\times$ $1^{\circ }$ spatial resolution using the Climate Data Operators (CDO) toolkit^37^. Trend estimation was performed using the Iteratively Reweighted Least Squares (IRLS) method^38^, which improves robustness in detecting long-term trends.

### Regular optimal fingerprinting (ROF) method

Additionally, a ROF method analysis was performed^25^ by using HadCRUT5 observations, and different CMIP5 model runs, implemented in Python^39^. This approach evaluates the influence of individual forcings to the total SAT change observed by HadCRUT5 to SAT fluctuations^10^. The ROF method has been successfully utilized in several key studies^10,17,25–27^ to detect and attribute the influence of specific forcings on SAT rise. The calculation process of ROF steps are outlined below:

The step begins by modeling by using HadCRUT observed temperature change ($y$) as regression of various model simulated forcing (such as GHG, Aaer, NAT) ($x_1, x_2, \ldots , x_n$), and ($\epsilon$, which is residual value):

$$\begin{aligned} y = \beta _0 + \beta _1 x_1 + \beta _2 x_2 + \ldots + \beta _n x_n + \epsilon \end{aligned}$$

Then the SAT values are arranged as matrix for computational efficiency. $X$ represents the different considered forcings matrix, $Y$ is the observed SAT vector, and $\beta$ the vector of coefficients to be estimated:

$$\begin{aligned} X= & \begin{bmatrix} 1 & x_{1,1} & x_{2,1} & \ldots & x_{n,1} \\ 1 & x_{1,2} & x_{2,2} & \ldots & x_{n,2} \\ \vdots & \vdots & \vdots & \ddots & \vdots \\ 1 & x_{1,m} & x_{2,m} & \ldots & x_{n,m} \end{bmatrix} \\ Y= & \begin{bmatrix} y_1 \\ y_2 \\ \vdots \\ y_m \end{bmatrix} \\ \beta= & \begin{bmatrix} \beta _0 \\ \beta _1 \\ \vdots \\ \beta _n \end{bmatrix} \end{aligned}$$

Thus, the regression can be written as $Y = X \beta + \epsilon$.

Then to calculate the coefficient associated with ($\beta$) we apply least squares approach to reduce the summation of the squared residuals of the HadCRUT observation and predicted SAT: $[ \beta = (X^T X)^{-1} X^T Y ]$

Then, we compute residual ($\epsilon$), these are the disparity of the HadCRUT5 observed SAT and predicted SAT. The model uncertainties are considered as the standard deviation of the obtained residuals ($STD\_residual$).

Next, estimation of confidence intervals (CI) at 95% of the ($\beta _i$) are computed as:

$$\begin{aligned} \text {CI} = [\beta _i - 1.96 \cdot STD\_residual, \beta _i + 1.96 \cdot STD\_residual] \end{aligned}$$

Then to assess the significance associated with different forcings estimations, we check whether the CI of different coefficients has zero. When zero is excluded from the CI, then the corresponding forcings are significant in terms of statistics in explaining HadCRUT SAT observational shifts.

### Calculation of the SPEI drought index

The Standardized Precipitation Evapotranspiration Index (SPEI) was calculated for individual SAT forcings following the methodology proposed by^23^. In this work, we employed the r1i1p1f1 ensemble for analyzing both surface air temperature (SAT) and precipitation, as SAT data corresponding to various forcings are exclusively accessible for this particular ensemble. Precipitation simulations from all the above models are utilized. The SPEI combines precipitation ($P$) and potential evapotranspiration (PET) to quantify moisture availability and its temporal variability. The steps for deriving SPEI are detailed below.

#### Potential evapotranspiration (PET)

Potential evapotranspiration ($PET$) was estimated as a function of surface air temperature ($SAT$) anomalies using an empirical formulation:

1 $$\begin{aligned} \text {PET} = k \cdot (\text {SAT} + c), \end{aligned}$$

where:

- $k = 0.0023$ is a scaling coefficient specific to the dataset.
- $\text {SAT}$ represents surface air temperature anomalies (in $^\circ \textrm{C}$).
- $c = 17.8$ is an offset constant ensuring positive values for PET.

This simplified approach balances computational efficiency and accuracy, aligning with the requirements of large-scale drought analysis.

#### Climate water balance

The climate water balance ($W$) represents the net subtraction between precipitation ($P$) and potential evapotranspiration ($PET$):

2 $$\begin{aligned} W = P - \text {PET}, \end{aligned}$$

where both $P$ and $PET$ are expressed in millimeters ($mm$).

#### Temporal aggregation of water balance

To capture cumulative drought effects over time, the climate water balance was aggregated using a rolling sum for a defined temporal scale ($n$):

3 $$\begin{aligned} W_{\text {rolling}}(t) = \sum _{i=t-n+1}^{t} W(i), \end{aligned}$$

where:

- $t$ is the current time step.
- $n$ is the chosen time scale (e.g., 12 months for annual SPEI).

This rolling sum accounts for the persistence of water deficits or surpluses over a given period.

#### Standardization of the climate water balance

The aggregated water balance ($W_{\text {rolling}}$) was standardized to transform it into a dimensionless drought index. Standardization was performed using:

4 $$\begin{aligned} Z = \frac{W_{\text {rolling}} - \mu }{\sigma }, \end{aligned}$$

where:

- $\mu$ is the mean of the aggregated water balance.
- $\sigma$ is the standard deviation of the aggregated water balance.

This standardization ensures comparability across different temporal scales and regions.

#### Conversion to the SPEI index

The standardized values ($Z$) were then converted into probabilities using the cumulative distribution function (CDF) of the standard normal distribution:

5 $$\begin{aligned} F(Z) = \Phi (Z), \end{aligned}$$

where $\Phi (Z)$ is the standard normal CDF.

Finally, these probabilities were mapped back to the standard normal distribution to derive the SPEI values:

6 $$\begin{aligned} \text {SPEI} = \Phi ^{-1}(F(Z)), \end{aligned}$$

where $\Phi ^{-1}$ represents the inverse standard normal distribution.

#### Application to forcing-specific SAT data

The above procedure was applied to individual and total SAT forcings, including anthropogenic (Ant), GHG, LU, Aaer, and NAT components. For each forcing:

1. $PET$ was computed using SAT anomalies.
2. The climate water balance ($W$) was calculated with observed precipitation.
3. SPEI values were derived at monthly and annual scales.

This approach provides a detailed assessment of the causal contributions of each forcing to drought conditions across Africa during the pre-industrial (1850–1900) and industrial (1955–2005) periods.

## Supplementary Information

Supplementary Information.

## Data Availability

The CMIP5 model datasets used in this study can be accessed at https://esgf-data.dkrz.de/search/cmip5-dkrz/. Observational temperature data from HadCRUT5 is available at https://crudata.uea.ac.uk/cru/data/temperature/. The Global Standardized Precipitation Evapotranspiration Index (SPEI) dataset was sourced from https://spei.csic.es/database.html.
