# Supplementary material for: Human-induced temperature rise is driving Africa towards drought-prone climatic conditions
Source: Sci Rep. 2026 Jan 3;16:630. doi: 10.1038/s41598-025-34010-6 (PMC12775511; doi:10.1038/s41598-025-34010-6)
Supplement: Supplementary file 1 — Supplementary Information. [file 41598_2025_34010_MOESM1_ESM.pdf]

Human-induced temperature rise is driving Africa towards drought-prone climatic conditions

Sl.No.	Model Name	Historical (1850-2005)					Future Projection (2006-2100)			
		Historical	AA	GHG	LU	NAT	RCP2.6	RCP4.5	RCP6.0	RCP8.5
1	bcc-csm1-1	Y	N	Y	N	Y	Y	Y	Y	Y
2	BNU-ESM	Y	N	Y	N	Y	Y	Y	N	Y
3	CanESM2	Y	Y	Y	Y	Y	Y	Y	Y	Y
4	CCSM4	Y	Y	Y	Y	Y	Y	Y	Y	Y
5	CESM1-CAM5	Y	N	Y	N	N	Y	Y	Y	Y
6	CNRM-CM5	Y	N	Y	N	Y	Y	Y	Y	Y
7	CSIRO-MK3	Y	Y	Y	N	Y	Y	Y	Y	Y
8	FGOALS_g2	Y	Y	Y	N	Y	Y	Y	N	Y
9	GFDL-CM3	Y	Y	Y	N	Y	Y	Y	Y	Y
10	GFDL-ESM2M	Y	Y	Y	Y	Y	Y	Y	Y	Y
11	GISS-E2-H	Y	Y	Y	Y	Y	Y	Y	Y	Y
12	GISS-E2-R	Y	Y	Y	Y	Y	Y	Y	Y	Y
13	HadGEM2-CC	Y	N	N	N	N	Y	Y	Y	Y
14	HadGEM2-ES	Y	N	Y	N	Y	Y	Y	Y	Y
15	inmcm4	Y	N	N	N	N	Y	Y	Y	Y
16	IPSL-CM5A-LR	Y	Y	Y	N	Y	Y	Y	Y	Y
17	IPSL-CM5A-MR	Y	N	Y	N	Y	Y	Y	Y	Y
18	MIROC-ESM	Y	N	Y	N	Y	Y	Y	Y	Y
19	MIROC5	Y	N	N	N	N	Y	Y	Y	Y
20	MPI-ESM-LR	Y	N	N	N	N	Y	Y	Y	Y
21	MRI-CGCM3	Y	N	Y	N	Y	Y	Y	Y	Y
22	NorESM1-M	Y	Y	Y	N	Y	Y	Y	Y	Y
	Total	22	10	18	5	17	22	22	20	22

Table S1 Table containing information about different models used in this study. Our study utilizes 158 CMIP5 simulations encompassing RCP26, RCP45, RCP60, and RCP85 scenarios. The historical simulations incorporate various forcings, with 'Y' indicating the inclusion of a particular forcing and 'N' denoting its absence. The forcings include anthropogenic aerosols (AA), well-mixed greenhouse gases (GHG), land-use change (LU), and natural forcings (NAT). All forcing information was sourced from the CMIP5 website: http://cmip-pcmdi.llnl.gov/index.html.). In this study, we utilized a single ensemble (r1i1f1p1) for both SAT and Precipitation because the SAT data for different forcings are only available for this specific ensemble. The precipitation simulations are used from all the above models.

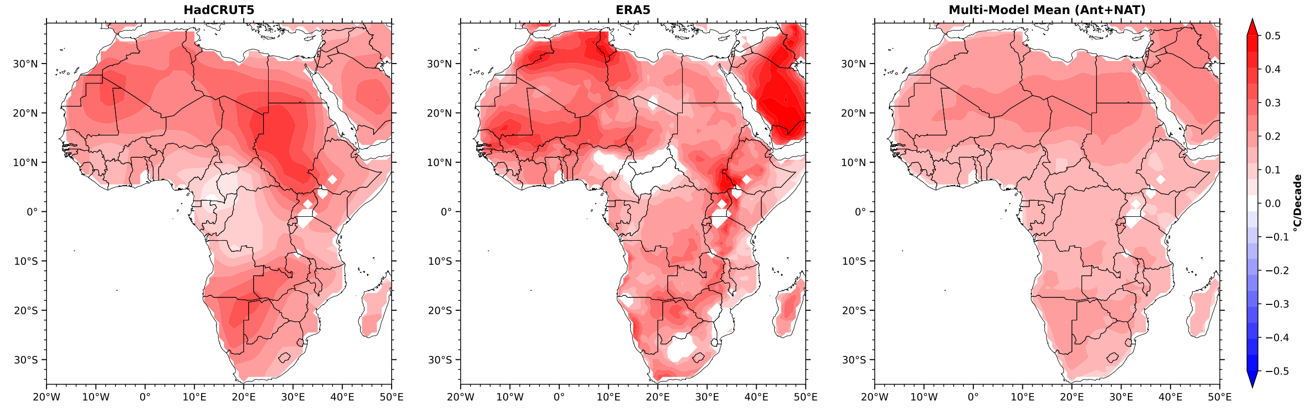

Fig. S1 Spatial Surface air temperature trends from HadCRUT5, ERA5, and multi-model mean (Ant+NAT). Temperature trends for the Industrial are depicted using the average of observational datasets like HadCRUT5, ERA5, and multi-model simulations. Regions lacking statistically significant changes are masked out.

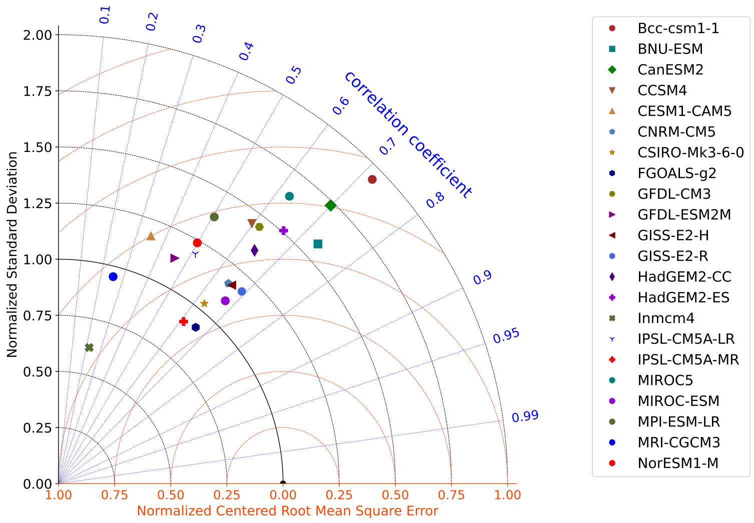

(a)

Fig. S2 Taylor diagram presenting evaluation of surface air temperature simulated by different models with respect to HadCRUT5 observations. The Taylor diagram illustrates the mean surface air temperature comparison among HadCRUT5, and individual CMIP5 models from 1955 to 2005. Solid lines represent standard deviations, while dotted lines indicate correlations.
